# 

**Published:** 1874-07

**Authors:** 


### *Directory for the Month of July.*

*Clinics.*—At Mercy Hospital, 3 P. M. Surgery.—Dr. Andrews. At the Central Dispensary, (239 W. Van Buren street), 3 P. M. Medical.—Dr. Bridge. At the Eye and Ear Infirmary, 2 P. M.—Dr. Holmes. At Hospital for Women and Children, 1.30 P. M. Gynæcological.—Dr. Thompson.

*Mondays, July 6th and 20th.*—Meeting of *Chicago Medical Society*, in the evening, at Gault House.

*Mondays, July 13th and 27th.*—Meeting *Chicago Association of Physicians and Surgeons*, in the evening, at Grand Pacific Hotel.

*Clinics.*—At County Hospital, 2 P. M. Surgical.—Dr. Bogue. Medical.—Dr. H. M. Lyman. At Mercy Hospital, 3 P. M. Gynæcological.—Dr. Roler.

*Clinics.*—At County Hospital, 2 P. M. Gynæcological.—Dr. Quine, 3 P. M. Ophthalmological.—Dr. Hotz. At Mercy Hospital, 3 P. M. Ophthalmological.—Dr. S. J. Jones. At St. Luke's Hospital, 8 A. M. Surgical.—Dr. Owens. At Central Dispensary, 3 P. M.—Dr. Bridge.

*Clinics.*—At Mercy Hospital, 3 P. M. Medical.—Dr. Merriman. At Central Dispensary, 2 P. M. Gynæcological.—Dr. Adolphus. Diseases of Chest. Dr. E. F. Ingals. At Hospital for Women and Children, 1.30 P. M. Medical. —Dr. A. H. Foster.

*Clinics.*—At County Hospital, 2 P. M. Medical.—Dr. Bevan. Surgical —Dr. Bogue. At Mercy Hospital, 3 P. M. Medical.—Dr. Nelson.

*Clinics.*—At Rush College, 2 P. M. Surgery.—Dr. Gunn. 3 P. M. Diseases of Nervous System.—Dr. Hay.

**TERMS, ALWAYS IN ADVANCE:** One copy, per annum.... $3 00

☞ Yearly subscriptions must begin with the January number.

☞ Persons who send subscriptions for the JOURNAL will please consider the first number received after subscription as a sufficient receipt for money sent.

☞ We cannot return rejected manuscripts.

CHICAGO:

W. B. KEEN, COOKE & CO., PUBLISHERS,

NOS. 113 AND 115 STATE STREET.

☞ **Back Nos. for 1872, '73 and '74 can be supplied at 30 cents each.**

